# The Effect of Holding Time on Dissimilar Transient Liquid-Phase-Bonded Properties of Super-Ferritic Stainless Steel 446 to Martensitic Stainless Steel 410 Using a Nickel-Based Interlayer

**DOI:** 10.3390/mi13111801

**Published:** 2022-10-22

**Authors:** Majid Hafizi, Masoud Kasiri-Asgarani, Mojtaba Naalchian, Hamid Reza Bakhsheshi-Rad, Filippo Berto

**Affiliations:** 1 Advanced Materials Research Center, Department of Materials Engineering, Najafabad Branch, Islamic Azad University, Najafabad, Iran; 2 Department of Chemical Engineering Materials Environment, Sapienza University of Rome, 00184 Rome, Italy

**Keywords:** microstructure, mechanical property, bonding temperature and time, transient liquid phase bonding, super-ferritic stainless steel, martensitic stainless steel

## Abstract

The dissimilar joining of martensitic and ferritic stainless steels have been developed that needs corrosion resistance and enhanced mechanical properties. In this study, the transient liquid-phase bonding of martensitic stainless steel 410 and super-ferritic stainless steel 446 was conducted with a nickel-based amorphous interlayer (BNi-2) at constant temperature (1050 °C) and increasing times of 1, 15, 30, 45, and 60 min. For characterization of the TLP-bonded samples, optical microscopy and scanning emission microscopy equipped with energy-dispersive X-ray spectroscopy were used. To investigate the mechanical properties of TLP-bonded samples, the shear strength test method was used. Finally, the X-ray diffraction method was used for microstructural investigation and phase identification. The microstructural study showed that the microstructure of base metals changed: the martensitic structure transited to tempered martensite, including ferrite + cementite colonies, and the delta phase in super-ferritic stainless steel dissolved in the matrix. During the transient liquid-phase bonding, the aggregation of boron due to its diffusion to base metals resulted in the precipitation of a secondary phase, including iron–chromium-rich borides with blocky and needle-like morphologies at the interface of the molten interlayer and base metals. On the other hand, the segregation of boron in the bonding zone resulted from a low solubility limit, and the distribution coefficient has induced some destructive and brittle phases, such as nickel-rich (Ni_3_B) and chromium-rich boride (CrB/Cr_2_B). By increasing the time, significant amounts of boron have been diffused to a base metal, and diffusion-induced isothermal solidification has happened, such that the isothermal solidification of the assembly has been completed under the 1050 °C/60 min condition. The distribution of the hardness profile is relatively uniform at the bonding zone after completing isothermal solidification, except the diffusion-affected zone, which has a higher hardness. The shear strength test showed that increasing the holding time was effective in achieving the strength near the base metals such that the maximum shear strength of about 472 MPa was achieved.

## 1. Introduction

Ferritic stainless steels have good resistance to stress corrosion cracking, cavity corrosion, and groove corrosion, especially in chloride environments. These types of steels are used in various applications, where corrosion resistance is more important than mechanical properties [1]. Low chromium grades (10.5 to 12.5 wt.% chromium) are used in applications such as exhaust systems that require normal corrosion resistance [2]. Medium and high chromium grades are used in highly corrosive environments. Super ferrite alloys are used in chemical and printing processes, where corrosion resistance is required in highly oxidized environments [3]. Martensitic stainless steels are iron–chromium alloys with 12–17 wt.% of chromium and have a sufficient amount of carbon, which, by rapid cooling from the austenitic region, gives a martensitic structure, which is why they are called martensitic steels, because they are created with the austenitizing and quenching. Super-ferritic stainless steels have excellent corrosion resistance compared to the martensitic and austenitic groups, but they cost more and have problems in construction. On the other hand, their use is limited to temperatures below 400 °C and this is due to being composed of brittle phases [4,5]. The dissimilar bonding of stainless steel due to the diversity of chemical composition, different physical and chemical properties, as well as the coefficient of thermal expansion in some very different cases, has caused it to become complicated [6,7]. Differences in thermal expansion coefficients can cause residual stresses in the joint and greatly reduce the performance of the part [8]. Welding processes, especially arc welding, have been common methods in joining various compounds of stainless steel. Ghosh et al. [9] investigated the dissimilar welding of 409 ferritic stainless steels and 316 austenitic stainless steels by metal–gas arc welding with 308L filler metal. They believed the combination is used in many industries, such as the radiation industry, shipbuilding, nuclear power plants, and the paper and food industries. These joints can have economically and technically acceptable performance. Sashank et al. [10], in their review of the dissimilar welding bonding of austenitic stainless steels to martensitic stainless steels, stated that this dissimilar bonding is very useful in nuclear reactors used at high temperatures. Based on this, it seems that the dissimilar combination of martensitic stainless steels with good mechanical properties along with ferritic/super-ferritic stainless steels with good corrosion-resistant properties can be achieved, where good corrosion and mechanical properties are combined. One needs to be used as an application.

In ferritic stainless steels, weld solidification cracking occurs in the final stages of solidification due to the combination of impurities and the separation of alloying elements, the formation of a thin layer of melt at the grain boundary, and strong mechanical constraints [11]. Cracking is often accompanied by solidification of the grain boundaries, with the greatest elemental separation occurring at low solidification temperatures [12]. When the initial phase is the solidification of ferrite, the sensitivity to the solidification of the weld is generally lower [13]. All ferritic stainless steels solidify as primary ferrites, which is why cracking is almost rare in this type of steel. The addition of alloying elements, such as titanium, niobium, and high levels of impurities, increases the likelihood of solidification cracks because separation during solidification can lead to the formation of thin melt layers with low melting temperatures along the grain boundary [14]. In addition, these alloys have an almost narrow solidification range that limits the amount of inhibition caused by shrinkage during solidification. Low-chromium alloys are less sensitive to welding cracks, while high-chromium alloys are usually welded under highly controlled conditions. In martensitic stainless steels, due to the presence of untempered martensite that occurs after welding, the sensitivity to hydrogen cracking may be high [15]. Solid-state bonding processes, one of which is transient liquid phase (TLP) bonding, can be used in dissimilar bonding [16] and specifically bond ferritic/super-ferritic and martensitic stainless steels and eliminate fusion welding problems. In this process, an interlayer containing melting-point depressant elements is placed between the adjacent surfaces of the joint, and the interlayer is heated above the liquidus temperature, the interlayer melts, and the melting point, reducing elements penetrating the base metals; after the bonding process is completed, the re-melting temperature of the interlayer increases [17]. The most important parameters in this process are temperature, time, interlayer thickness, the chemical composition of the interlayer, and the bonding atmosphere, each of which affects the quality of the bonding [18]. In many studies on TLP bonding, due to the solubility of the element in nickel filler metals and specifically in the amorphous state, brazing and transient liquid-phase bonding of similar and dissimilar alloys is common [19,20,21].

In recent years, extensive research has been conducted in the field of bonding similar and dissimilar metals in the process of transient liquid-phase bonding on different groups of stainless steels. For example, Sadeghian et al. [22] evaluated the effect of bonding time and homogenization on the bonding of 304 austenitic stainless steel with a cobalt alloy interlayer using the transient liquid-phase method on its microstructure and mechanical properties. Isothermal solidification was completed at 1180/30 °C. Abdolvand et al. [23] investigated the effect of bonding time of super-duplex stainless steel SAF2507 to 304 austenitic steel using the transient liquid-phase method with a BNi-2 intermediate layer at 1050 °C. Experimental results showed that at a constant temperature, the width of the non-isothermal solidification zone decreases with the increasing holding time. In addition, with the increasing shear strength time, isothermal solidification is completed in 45 min. Jafari et al. [24] evaluated the dissimilar bonding of martensitic stainless steel 420 to super-duplex stainless steel 2507 using the transient liquid-phase bonding process. Their result exhibited that bonding at 1050 °C temperature and different holding times causes different microstructural regions along the joint seam, which has a significant effect on the micro-hardness and mechanical properties. The completion of isothermal solidification at 60 min resulted in the maximum shear strength due to the single-phase γ-Ni structure in the isothermal solidification zone.

Super-ferritic stainless steel is used for exhaust applications but does not have good mechanical properties, so the dissimilar welding of this material to martensitic stainless steels that have more beneficial mechanical properties can increase the supply and applicable ability and lifespan of this steel. The dissimilar TLP of super-ferritic stainless steel 446 to martensitic stainless steel has been seldom investigated in the literature. So, the aim of this study in transient liquid-phase bonding of super-ferritic 446 stainless steel to martensite 410 is to investigate the effect of holding time at a constant temperature on the microstructure of the bonded area and the mechanical properties of the joint. Given that this dissimilar bonding has not yet been studied with welding and solid-state processes, its microstructural attractiveness could be a new window into the dissimilar bonding of martensitic and ferritic stainless steels.

## 2. Experimental Method

### 2.1. Materials and Bonding

In this research, super-ferritic stainless steel 446 (SFSS 446) sheets and martensitic stainless steel 410 (MSS 410) sheets with a thickness of 5 mm were used as base materials, the chemical compositions of which are given in Table 1. For the bonding operation, small parts with a size of 10 × 10 mm^2^ were prepared by electrical discharge machining wire cutting, and after that, the samples were washed in a thinner solution of 10,000 to completely remove oil and grease contaminants. All samples’ surfaces were cleaned with No. 60 sandpaper to remove the surface oxide layer. The adjacent bonding surface was sandpaper with emery No. 80 to 1200.

**Figure 1:**
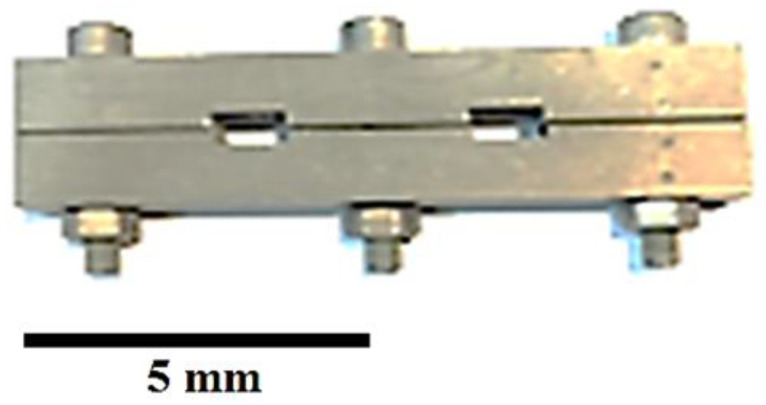
The fixture used to transient liquid phase bond the SFSS 446 and MSS 410.

To perform the bonding process, it is necessary to use the interlayer to act as a bonding agent. Based on studies [19,20,21,22,23] and field research, the BNi-2 interlayer is used as one of the most important and widely used interlayers in various industries for bonding and repairing. Hence, 50 μm thick amorphous BNi-2 foil (MBF-20) was selected as the interlayer, the chemical composition of which is given in Table 2. The interlayer foils were also cut to 10 × 10 mm. The interlayer and samples were then degreased with an ultrasonic (WUC-D10H, Wisd Laboratory Equipments, Wertheim, Germany) device containing acetone. The initial degreasing operation was performed for one hour to ensure the cleanliness of the samples. After degreasing, the samples were kept in an alcohol container. A suitable fixture was used to perform the bonding operation. The main function of the fixture is to keep the specimens firmly in place during heating until the bonding temperature is reached and to prevent the flow of the molten interlayer material. Therefore, it must be suitable to maintain its properties well at high temperatures. Among the proposed options, austenitic stainless steels are among the most common and inexpensive materials that can be used. Therefore, austenitic stainless steel 316 has been used as a fixture. For bonding, the interlayer was placed between two samples of super-ferrite 446 stainless steel and martensitic stainless steel 410. Then, approximately 0.3 MPa pressure was applied to the samples using the screws installed on them. Figure 1 shows the holder used to connect the transient liquid phase of super-ferritic 446 and martensitic 410 stainless steels. Bonding was performed in a vacuum furnace with a relative vacuum of 10-4 torr at a bonding temperature of 1050 °C with holding times of 1, 15, 30, 45, and 60 min. A heating rate of 15 °C/min was used. After the coupling cycle was complete, the samples were cooled in the furnace at an approximate rate of 10 °C/min.

### 2.2. Bonding Characterization

#### 2.2.1. Microstructural Characterization

To investigate the microstructure of the bonded specimens, the specimens were cut perpendicular to the joint after the bonding process and mounted with epoxy resin. Grade 80 to 4000 sandpaper was used to prepare the metallographic samples. They were then thoroughly cleaned with water and alcohol to prepare for the initial polishing step. Alumina with a grain size of 1 μm and a Struers polishing pad (Denmark) was used for initial polishing. Then, the samples were used alumina with a grain size of 0.05 μm for the final polishing. For optical metallography, it is necessary that the samples prepared from the previous step are well etched. Two steps were used to perform the etching operation. First, the samples were etched with Murakami solution with a combination of 10 g KOH + 10 g K_3_Fe(CN)_6_ + 100 mL H_2_O for 60 s, and a solution with a combination of ethanol 100 mL + hydrochloric acid 100 mL + copper chloride 2 gr was used for 10 s. The etched samples were washed well with water and alcohol and kept in a glass chamber for the photographic stage under a light microscope. An Axiotech Zeiss optical microscope (ZEISS, Munich, Germany), equipped with Image J image analysis (National Institutes of Health, Bethesda, MD, USA) was used to examine the microstructure of samples including joints and base metals. These analyses included measuring the grain size of base metals, measuring the bonding area, and measuring the diffusion-affected zone. The scanning electron microscope (SEM; JEOL JSM-6380LA, Tokyo, Japan) equipped with energy dispersive X-ray spectrometer (EDS; JEOL Ltd., Akishima, Japan), in order to accurately determine the phases formed at the bonding position and to study the concentration changes at the bonding position was used. To confirm the phases formed in the bonding region, the X-ray diffraction method with Cu-Kα conditions, λ = 1.54 was used.

#### 2.2.2. Mechanical Characterization

Hardness changes at the bonding position were investigated using the Vickers hardness tester (HMV-2T E, Shimadzu, Kyoto, Japan). For this purpose, a load of 50 g and a holding time of 10 s were used. Hardness changes in the bonding area, the interface, and the diffusion affected zone were measured. Due to the fact that bonding materials in the transient liquid-phase bonding method are a subset of the brazing processes, the shear strength test has been used to determine the bond strength. This test is performed according to the standard DASTM1002-05 with a strain rate of 1 mm/min. In order for the load to be applied precisely to the TLP-bonded sample, it is necessary to use a holder to convert the tensile stress into shear stress.

**Figure 2:**
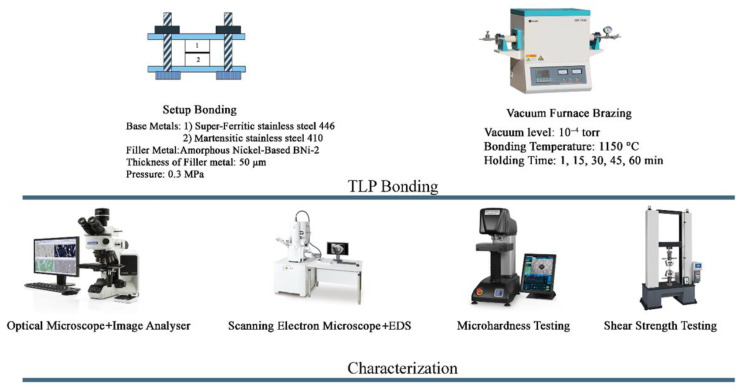
Schematic of experimental setup of the diffusion bonding processes of super-ferritic and martensitic stainless steel by BNi-2 amorphous foil.

For this purpose, the TLP specimens with 10 × 10 × 10 mm^3^ dimensions were placed inside the special holder [25]. Then, the holding was placed in the jaws of the tension device, and the load was applied. This was performed using the Wolpert-FM2750 (Japan) device. The evaluation of shear strength on each series of samples was repeated three times. Figure 2 shows the schematic of experimental setup of the diffusion bonding processes of super-ferritic and martensitic stainless steel by BNi-2 amorphous foil.

## 3. Result and Discussion

### 3.1. Microstructure of Base Metals

**Figure 3:**
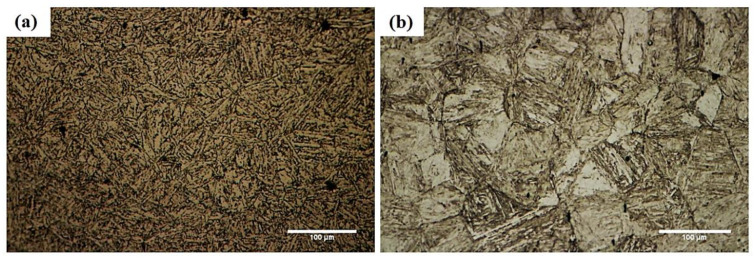
The optical microscopy of the microstructure of martensitic stainless steel410: (**a**) as-received and (**b**) under 1050 °C/60 min conditions.

Figure 3a shows the microstructure of martensitic stainless steel 410 in the rolled condition. According to a microstructural study, martensitic stainless steel 410 comprises a martensitic matrix with trace levels of residual austenite and M23C6 carbides. Martensitic stainless steel has a grain size of around 60 μm and a hardness of roughly HV490. Martensitic stainless steels must undergo heat treatment in order to attain the desired mechanical and corrosion characteristics for a given application [26]. Figure 3b shows optical microscopy of the microstructure of martensitic stainless steel at a bonding condition of 1050 °C/60 min. This temperature is within the range of martensitic stainless steel’s austenitizing temperature (925–1070 °C). Therefore, there is a sufficient chance for the transition of martensite to tempered martensite, including ferrite + cementite colonies, owing to the fact that cooling is also carried out within the furnace under equilibrium conditions. Additionally, part of the carbon and alloy components between the austenite and carbide phases result in dissolving the carbides during the joining process. According to Pickering [27], martensitic transformation is affected by carbon and other carbide-forming elements when heat treatment is applied above the temperature at which carbides dissolve. This is because the martensitic transformation’s range and start and finish temperatures are both lowered.

**Figure 4:**
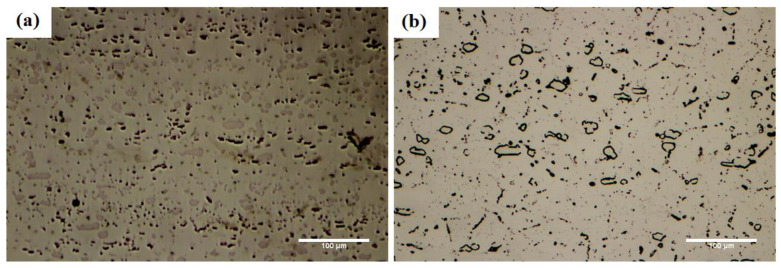
The optical microscopy of the microstructure of super-ferritic stainless steel 446: (**a**) as-received and (**b**) 1050 °C/60 min conditions.

The microstructure of rolled super-ferritic stainless steel 446 is seen in Figure 4a. Analysis of a portion of the alloy’s matrix revealed that it contains significant amounts of iron, along with chromium, nickel, molybdenum, and niobium, that helped form the ferrite solid solution. The alloy is made more stable by adding a significant quantity of chromium (27 wt.%), and the particles of the sigma phase are dispersed in two blocky and plate morphologies. For analyzing the chemical composition and the sigma morphologies, EDS point analysis has been used. There are two morphologies of sigma-phase particles, and the chemical analysis of these particles is presented in Table 3. It is obvious that these particles include considerable quantities of chromium, which have been deposited together with iron, nickel, and niobium. The two morphologies are essentially the same. The amount of iron from the deposits is typically 27 wt.% (in as-received condition) and increases to 70 wt.% (at 1050 °C/60 min bonding condition), which indicates the tendency to be removed and dissolved in the matrix. Holding at the joining temperature has caused a significant amount of chromium in these phases to diffuse into the matrix through the solid-state (Figure 4b).

### 3.2. The Microstructure of the Bonded Area

**Figure 5:**
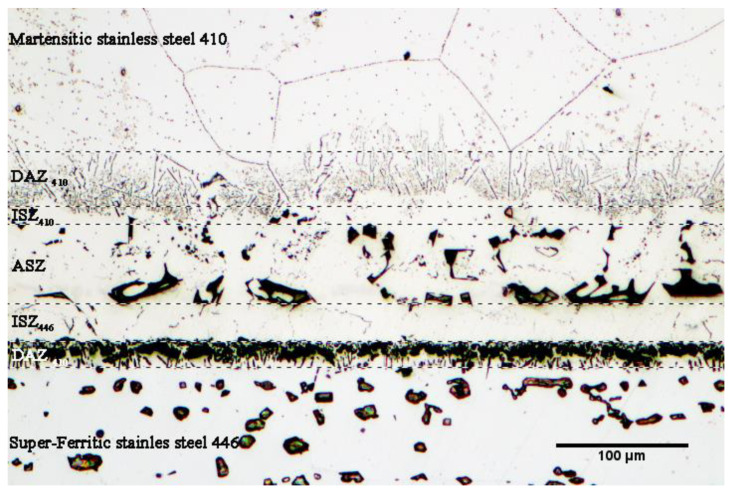
The optical microscopy of TLP-bonded sample at 1050 °C/1 min condition.

Figure 5 shows the microstructure of the bonded sample at 1050 °C/1 min. It is evident that the top is made of martensitic stainless steel 410 with extremely precise graining, the bonding region is in the center, and the bottom is made of super-ferritic stainless steel 446. The chemical composition’s slope brings on the microstructural gradient along the seam. The bonded area may be separated into the following divisions based on the microstructural changes:

(1) Martensitic stainless steel 410, (2) Diffusion-affected zone on the side of the martensitic stainless steel 410 (DAZ_410_), (3) Isothermal solidification zone on the side of the martensitic stainless steel 410 (ISZ_410_), (4) Athermal solidification zone (ASZ), (5) Isothermal solidification zone on the side of super-ferritic stainless steel 446 (ISZ_446_), (6) Diffusion-affected zone on the side of super-ferritic stainless steel 446 (DAZ_446_), and (7) Super-ferritic stainless steel 446.

**Figure 6:**
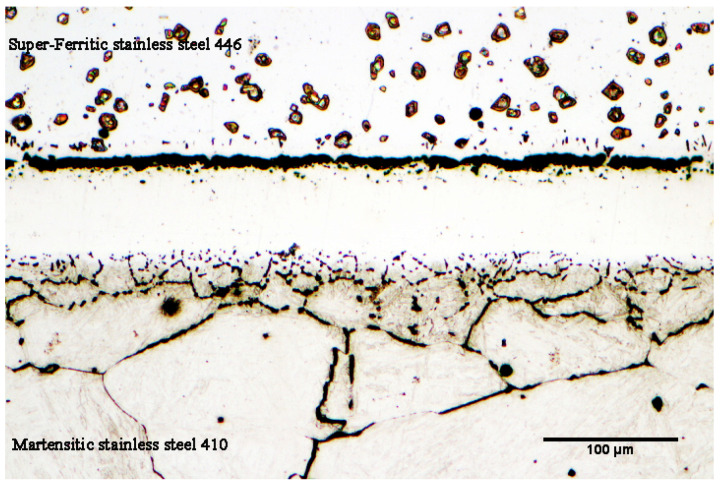
The microstructure of joint interface of the TLP-bonded sample at 1050 °C/60 min condition.

The research [28,29,30] indicates that there are four steps that make up the transient liquid-phase bonding process: (1) heating the joint assembly to the bonding temperature, (2) melting the interlayer and ensuring that its chemical composition is uniform throughout the joint, (3) dissolving the substrate metals to achieve thermodynamic equilibrium at the solid/melt interface, and (4) isothermal solidification of the mixture of the interlayer melt and the substrate’s dissolved portion. When the joint assembly is heating to a bonding temperature of 1050 °C, part of the melting point depressant is diffused to super-ferritic 446 and martensitic 410 stainless steels. The diffusion rate of the melting point depressant severely depends on the solubility and diffusion coefficients of both substrates. Diffusion of the melting-point-depressant element with a higher diffusion coefficient (boron) results in increasing the concentration beyond its solubility limit in iron. Because of its low atomic diameter (1.17 Å), strong activity, and greater efficacy in lowering the melting point of elements, boron should be emphasized as being more significant than other melting-point-depressant elements. It is noted that the diffusion of boron takes place at crystallographic defects such as grain boundaries, stacking faults, slip bands, and dislocations at low temperature, but bulk diffusion from the grain itself happens at high temperature. Gale et al. [28] examined the influence of boron’s diffusion in nickel substrates (Inconel 738 superalloy) using the nickel–boron phase diagram. They asserted that the solubility limit of boron in pure nickel in the eutectic temperature range (1093–1095 °C) is around 0.3 at.% according to the binary phase diagram of nickel–boron. Because the transient liquid-phase bonding process is based on the diffusion process, the binary nickel–boron phase diagram states that when boron diffuses through the molten interlayer, it initially collects at the interface between the interlayer and the base metal [29,30,31]. Given that nickel has a maximum solubility of roughly 0.3 at.% for boron, the production of inter-metallic compounds occurs when boron builds up to a level over the solubility limit. The intermetallic complex Ni_3_B is one of the most frequent compounds generated in the common phase of the substrate/base metal (nickel). The same can be said about this issue of the high carbon martensitic stainless steel 410 and low carbon super-ferritic stainless steel. Stainless steels are abundant in chromium and carbon components in this assembly. The highest solubility of boron in pure iron, which is very tiny, is around 0.005 wt.%, according to the binary iron–carbon phase diagram. However, by adding about 30 percent by chromium weight, boron’s solubility in iron rises and reaches about 0.0589 wt.% [32]. As a result, the iron matrix dissolves more boron (based on the ternary diagram of iron–chromium–boron [33]). The interaction of boron with iron results in the creation of the compounds Fe_2_B and FeB, as seen by the binary phase diagram of iron–boron. Chromium, an element that stabilizes the ferrite phase, contributes around 30 wt.% to the ferritic structure of super-ferritic stainless steel 446, making it super-ferritic. The stability of the austenite phase is caused by boron’s penetration into the ultra-ferritic middle layer of the stainless steel, which is supported by the iron–boron binary phase diagram. Consequently, the area from the diffusion-affected zone to the interface of interlayer/super-ferritic stainless steel 446 region has an austenitic structure. It is notable that the diffusion of boron and the presence of carbon in base metals causes deposits to accumulate in the vicinity of the joint that impedes grain coarsening. Due to this, transient liquid-phase bonding is ideal for joining steels that are susceptible to grain coarsening in the heat-affected zone (in fusion welding). Figure 6 depicts an optical microscopy photograph of the microstructural bonding zone under the conditions of 1050 °C/60 min. It can be seen that the adjacent area of the joint has a very low grain growth rate when compared to base metals. Secondary-phase compounds begin to develop at the interface when the joint assembly is heated above the solidus and liquidus temperatures.

**Figure 7:**
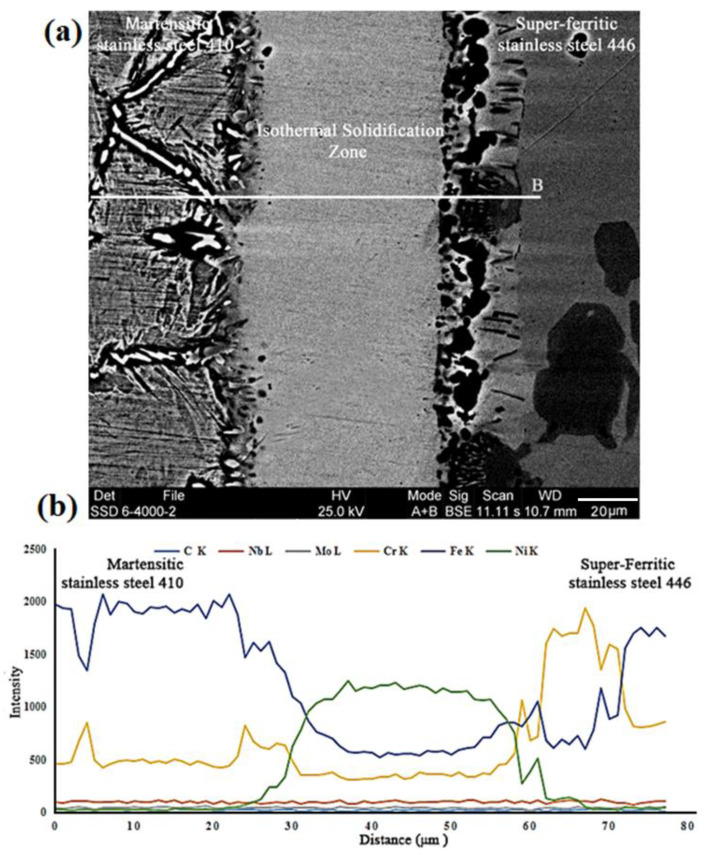
(**a**) The SEM micrograph and (**b**) line scans across the TLP bonded sample at 1050 °C/60 min condition.

When the interlayer reaches its solidus temperature (969 °C), it begins to melt, and from that point on, a greater rate of diffusion into the base metals from solid to molten state commences. The whole interlayer melts when the temperature rises to the liquidus temperature (1024 °C). As a result, boron diffuses to the base metals more quickly. In this study, an interlayer with an amorphous structure that is extremely similar to the eutectic composition of the Ni–B–Si phase diagram was used. A strong chemical composition gradient between the molten interlayer, which is rich in nickel, boron, and silicon, and the nearby base metals, which are rich in iron and chromium, happens when the interlayer melts fully and fills the joint. Based on what was previously said, boron, which is abundant in the interlayer, is the most significant element vital in the diffusion and production of the chemical composition gradient and the microstructure gradient. The simple segregation and quick diffusion of boron are the primary causes of this issue. This slope of the chemical composition results in the production of various microstructures along the bonding zone. The law of mass balance states that the melt and solid must follow thermodynamic equilibrium. Because the melt of the interlayer is rich in boron and the solid (base metal) contains a relatively small quantity of boron, which diffuses into the base metal during heating, part of the basic metals consequently dissolve in the melt of the interlayer. As a result, more iron and chromium, as well as other basic metal elements, enter the molten zone. Dissolution denotes this step of the transient liquid-phase bonding process. The width of the molten interlayer is increased throughout the dissolution stage, and the quantity of dissolution rises with time. The solidified joint has an average thickness of 72 μm, compared to the utilized interlayer of 50 μm, which shows that the interlayer has a significant dissolution of base metals in the molten interlayer. Base metals continue to dissolve until the liquid and solid chemical composition reach liquidus and solidus composition, respectively; in other words, the width of the molten interlayer achieves its maximum value. The liquid and solid follow the liquidus and solidus lines in the equilibrium phase diagram after thermodynamic equilibrium has been reached. The fourth step of the transitory liquid phase bonding, known as isothermal solidification, starts after thermodynamic equilibrium is reached. The main distinction between brazing and transient liquid phase joining is that they aid in forming a complete bonding by maintaining the assembly at a consistent temperature throughout the isothermal solidification stage. Because the process of diffusion from the molten state to the solid-state happens faster than the solid-state processes, the melting-point-depressant element (the faster element, boron) enters the base metals during isothermal storage. The melting temperature of the melt rises as elements that have a lower melting point than the melt interact with the base metals (the so-called liquidus temperature). Isothermal solidification begins as soon as the molten interlayer’s liquidus temperature reaches the bonding temperature [34]. As a result, the bonding temperature and the isothermal solidification temperature are not always identical [35]. The melting point rises initially along the solid/melt boundary with the diffusion of melting-point-depressant elements, which is where the first nickel-rich solid phase dendrites with a face-centered cubic crystal structure appear. These dendrites develop epitaxial on the base metal grains with a crystal direction that is parallel to the crystal direction of base metals, progressing towards the bonding centerline without the requirement for nucleation. These nickel-rich dendrites can only solve some amounts of elements, so, a little boron, chromium, silicon, iron, and other elements solved it and more amounts were rejected from the head of those. This phenomenon results in micro-segregation and element-rich residual liquid immediately at the solid/liquid interface which has effect on the mechanical properties of specimens. The volume of the melt is lowered when the melting depressant elements enter the base metals, and eventually, the interlayer melt solidifies entirely. The electron microscopy and EDS linear analysis along the bonded zone are shown in Figure 7. It is evident that no particles with destructive pseudo-eutectic morphology are present in the joint centerline or in the area around the joint, which is evidence that the isothermal solidification process at the joining condition of 1050 °C/60 min has been successfully completed. The EDS line analysis shows that the distribution of alloy elements is uniform at the bonding zone. However, when base metals are approached, the slope abruptly changes as a result of the precipitation and buildup of alloy elements. The concentration gradient’s secondary phases reach their highest point. The dissolution of base metals in the molten interlayer is indicated by the presence of elements such as iron, niobium, molybdenum, and an increase in the concentration of chromium in the bonding area. Their presence will significantly aid in raising the mechanical properties and remelting temperature in high-temperature applications.

**Figure 8:**
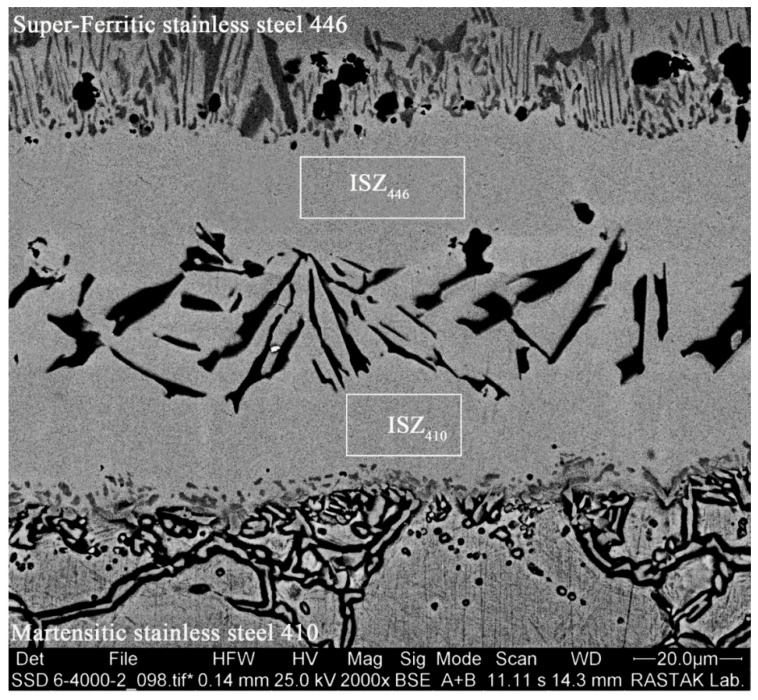
The SEM microscopy of a bonded sample at 1050 °C/15 min condition.

If the isothermal solidification process has not been completed and the amount of boron in the nickel matrix of the bonding zone is less than the solubility of boron (0.3 at. %), then the process of the diffusion of elements that lower the melting point (particularly boron) has not completed. So, the driving force of cooling from the bonding temperature to room temperature causes the remaining melt in the joint centerline to be solidified, which causes the remaining melt to follow the phase diagrams of nickel–silicon–boron [36] and become nickel–chromium–boron [37]. Figure 8 shows the SEM microscopy of the bonded sample under 1050 °C/15 min conditions. A number of compounds with plate and blocky morphologies with continuous distribution are seen, with the majority of them concentrating in the joint centerline, in contrast to the jointed sample under complete isothermal solidification conditions (1050 °C/60 min). Due to the segregation of alloy elements after solidification from the joining temperature to room temperature, these compounds seemed to be the site of accumulation. The isothermal solidification zone on the side of super-ferritic stainless steel 446 (ISZ446) and the isothermal solidification zone on the side of martensitic stainless steel 410 (ISZ410) are located on both sides of these intermetallic compounds. The area EDS analysis of these zones is shown in Table 4. It is obvious that these two areas are rich in nickel and are chemically identical to the sample’s whole isothermal zone at 1050 °C/60 min. According to the ternary phase diagrams of nickel–silicon–boron and nickel–chromium–boron, the initial phase that forms from the solid/melt interface by epitaxial growth is the nickel-rich pro-eutectic phase, which is formed through the diffusion of boron from the liquid state to base metals. The formation of the dendritic structure from the solid/liquid common phase and the rejection of the melting-point-depressant elements (particularly boron) results in the concentration of the melting-point-depressant elements in the residual melt increasing significantly [38]. Due to the high concentration of the element that lowers the melting point present, which causes it to combine with all of the alloy elements in the ternary and binary eutectic melts, this non-equilibrium segregation of elements causes the chemical composition of the melt to move away from the equilibrium state and move toward the eutectic state. Due to the high concentration of nickel and the lower Gibbs free energy of nickel boride, this process, known as a binary eutectic reaction, leads to the formation of a solid solution rich in nickel together with nickel-rich boride in the next stage. The residual melt becomes more enriched with k < 1 as a result of the development of primary dendrites. During stage I, the poor distribution coefficient of boron in nickel (0.008 based on the binary phase diagram of nickel–boron [39]) and the low solubility of boron in nickel (0.3 at.% based on the binary phase diagram of nickel–boron) result in the rejection of boron in the nearby melt and enriched at a location where the eutectic line crosses Ni_3_B. The eutectic line is followed throughout the solidification process such that the solid solution and nickel boride precipitate from the melt at the same time: L→γ-Ni + Ni_3_B + L.

**Figure 9:**
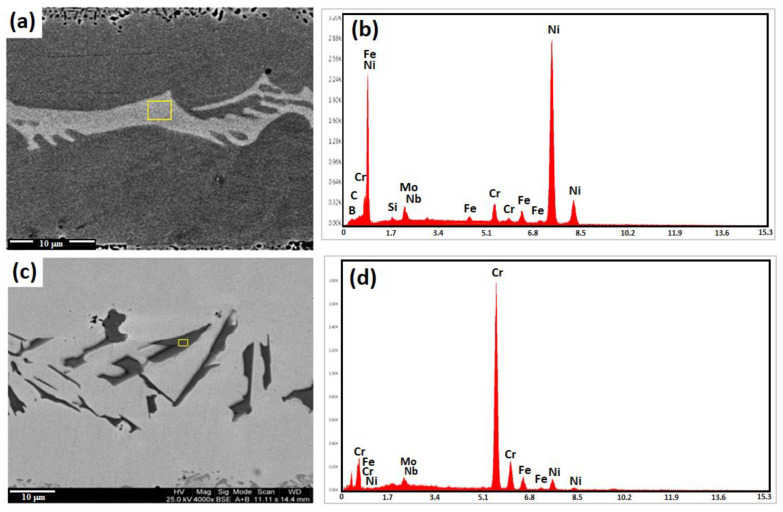
(**a**) The SEM micrograph and (**b**) EDS spectrum of nickel-rich boride in centerline joint of the TLP-bonded sample at 1050 °C/60 min condition; (**c**) the SEM micrograph and (**d**) EDS spectrum of chromium-rich boride in the centerline joint of the TLP-bonded sample at 1050 °C/15 min condition.

Figure 9a,b show the scanning electron microscopy of nickel-rich boride combined with an EDS spectrum, and Table 5 lists the chemical composition of this boride. In addition to nickel, another element that is rejected into the residual melt by the developing dendrites is chromium, which is strongly reactive with boron. The residual melt rose significantly in the earlier stages as a result of the limited solubility of chromium in nickel and nickel boride (10.11 and 18 at.%, respectively [40]). At this point, the conditions are favorable for the creation of an intermetallic compound of chromium and boron. Chromium-rich boride is visible in the bonded sample under bonding conditions of 1050 °C/15 min in Figure 9c,d, which is an image taken with a scanning electron microscope. It is obvious that non-equilibrium solidification caused these particles with random shapes to become solidified [41]. These particles’ EDS analysis reveals that the boride is mainly composed of chromium, with few additional alloying elements.

**Figure 10:**
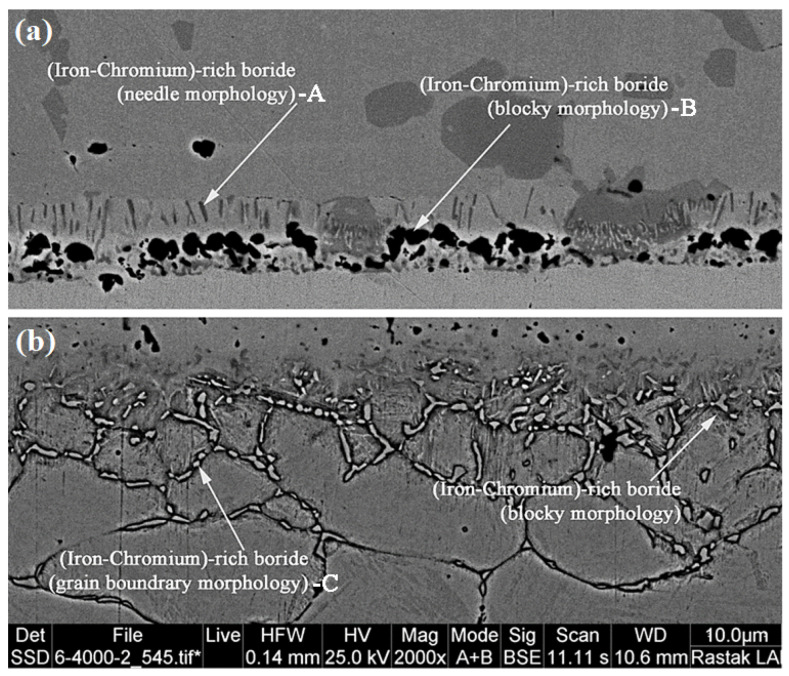
The microstructure of diffusion-affected zone on the side of: (**a**) super-ferritic stainless steel 446 an (**b**) martensitic stainless steel 410.

As was already indicated, the development of secondary-phase intermetallic compounds in the joint may be effectively induced by the solid-state diffusion of melting-point-depressant elements into base metals during heating [42]. These elements are diffused into the base metals at the onset of the isothermal solidification process, which is governed and influenced by substances that lower the melting point. The fact that they exist and have distinct morphologies from the base metal phases may be a sign that the isothermal solidification process is continuing and will eventually be finished. These secondary phases are still present close to the bonding interface, even if the conditions are ideal for complete isothermal solidification (1050 °C/60 min). It should be noted that when bonding temperatures are held for longer periods of time, a layer of secondary phases that had been continually forming during heating is disrupted, and a path is created for the diffusion of melting-point-depressant elements. According to Naalchian et al. [43] in their study on the dissimilar joining of transient liquid phases of cobalt-based superalloys X-45 and FSX-5414, nickel-rich carbo-boride deposits along the interface while heating up to the joining temperature have secondary phase compositions. A new generation of secondary-phase carbo-borides rich in cobalt, chromium, tungsten, and molybdenum deposits is created as a result of the production of the interlayer melt and isothermal holding in the dissolved molten interlayer. Because these boron-rich phases serve as a sink to absorb boron and accelerate the isothermal solidification process, it smooths out the melting-point-depressant element’s diffusion to base metals [44]. The diffusion-affected zones of super-ferritic stainless steel 446 and martensitic stainless steel 410 at a bonding condition of 1050 °C/30 min are shown in Figure 10 using a scanning electron microscope. Figure 10a,b make it evident that the morphologies developed completely differently. It is possible to see dark masses with needle-like deposits on the side of super-ferritic stainless steel 446.

**Figure 11:**
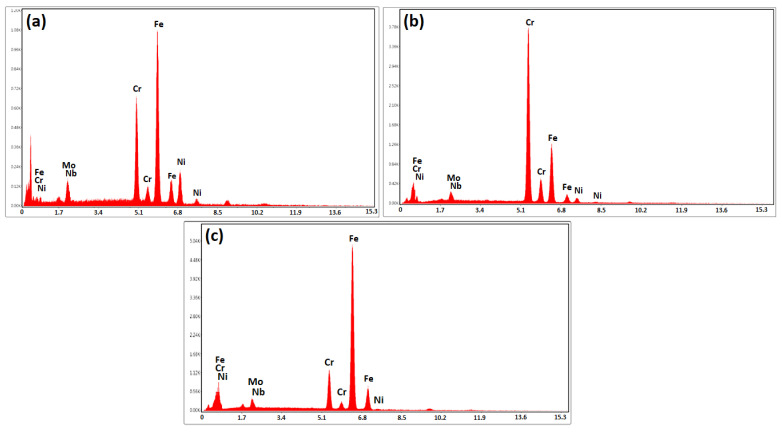
The EDS analysis of secondary-phase precipitations: (**a**) Point A, (**b**) Point B, and (**c**) Point C in Figure 10.

The EDS analysis of dark masses in Figure 11a reveals that they are rich in iron, chromium, and niobium, with minor levels of nickel. These precipitations have significantly dedicated the secondary phase in the diffusion-affected zone. Due to the considerable quantity of chromium in this alloy, it was predicted that the needle phases on the side of super-ferritic stainless steel 446 would be rich in chromium (Figure 11b). The condition is different, and most of the secondary phases develop discontinuously at the grain boundaries and are needle-like within the grains in the area where martensitic stainless steel 410 has diffused. These precipitations have a high concentration of iron, chromium, and niobium, according to an EDS point analysis (Figure 11c). Thus, these secondary-phase compounds develop as carbo-borides rich in alloying elements when boride-forming elements are present close to the carbon of the base metals [45].

### 3.3. Mechanical Properties

**Figure 12:**
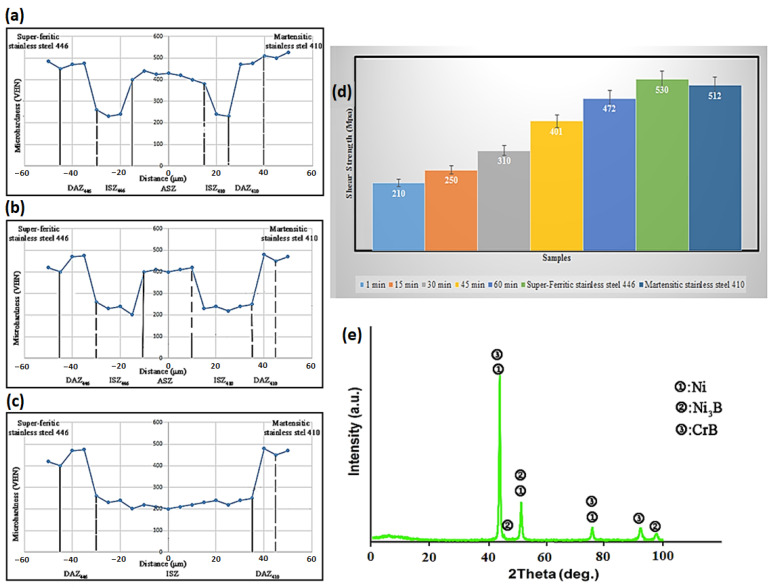
Hardness profile across the joint TLP-bonded region under constant temperature 1050 °C by increasing time: (**a**) 1 min, (**b**) 30 min, and (**c**) 60 min. (**d**) Shear strength of base metals and TLP-bonded metals under constant temperature of 1050 °C by increasing time. (**e**) The XRD pattern of TLP-bonded fracture surface under 1050 °C/15 min conditions.

Microhardness testing is one of the most significant procedures that is often used for the semi-quantitative assessment of chemical composition distribution and, ultimately, microstructural homogeneity. The findings of the microhardness test for the non-isothermal solidification samples under bonding conditions of 1050 °C/1 min and 30 min are shown in Figure 12a–c, as are the results for the completed isothermal solidification sample at 1050 °C for 60 min of bonding. Since eutectic particles are hard and brittle, their presence in the joint centerline of samples with non-isothermal solidification has enhanced the hardness. These eutectic microconstituents serve as preferred locations for crack nucleation [46]. The joint is made free of intermetallic compounds by increasing the holding time, or, in other words, by completing the isothermal solidification [47,48,49,50,51,52]. It is evident that the elimination of the diffusion-affected zone on the side of martensitic stainless steel 410 greatly lowered the microhardness peak. One of the key findings of the microhardness behavior of the samples is that, once isothermal solidification is complete, the base metals’ hardness is reduced, and the joint size is dramatically increased with an increase in holding time [53,54,55,56,57,58,59]. The joint seam thickness grows from 50 μm to 72 μm. The shear strengths of the jointed samples under various situations are shown in Figure 12d. Due to the presence of intermetallic compounds, such as boride compounds rich in chromium and nickel, the samples that joined quickly had the lowest degree of strength. The shear strength of the samples may be increased by prolonging storage at a constant temperature, eliminating intermetallic compounds from the joint centerline and establishing a fully isothermal solidification zone [60,61,62,63,64,65,66,67]. The shear strength tests’ findings support the joint region’s microstructural analyses. The jointed sample’s fracture surface’s X-ray diffraction pattern under the condition of 1050 °C/15 min is shown in Figure 12e. As expected by the microstructural investigations, the existence of solid solution phases rich in nickel, boride phases rich in chromium (CrB), and nickel-rich phases (Ni_3_B) are shown in this figure. This finding is also corroborated by Tazikeh et al. [49].

## 4. Conclusions

In this study, the transient liquid-phase bonding of martensitic stainless steel 410 and super-ferritic stainless steel 446 has been carried out using a nickel-based amorphous interlayer (BNi-2) at a constant temperature (1050 °C) and increasing the time by 1, 15, 30, 45, and 60 min. The microstructural and mechanical properties of TLP bonding were exhibited as follows:

1. The microstructure of martensitic stainless steel 410 in the rolled condition changes from martensite to tempered martensite, including ferrite + cementite colonies, owing to the fact that cooling is also carried out within the furnace under equilibrium conditions.
2. Super-ferritic stainless steel 446 is made more stable by adding a significant quantity of chromium (27 wt.%), and the particles of the sigma phase are dispersed in two blocky and plate-like morphologies. By increasing the holding time, the amount of iron from the deposits is typically 27 wt.% (in the as-received condition) and increases to 70 wt.% (at 1050 °C/60 min bonding condition), which indicates the tendency of iron to be removed and dissolved in the matrix. Holding at the joining temperature has caused a significant amount of chromium in these phases to diffuse into the matrix through the solid state.
3. During the heating of transient liquid phase bonding, the diffusion of boron to base metals and its interaction with iron and chromium results in the creation of iron–chromium-rich boride compounds.
4. When the isothermal solidification is not completed, intermetallic compounds such as Ni_3_B and CrB/Cr_2_B present as a network at the bonding centerline. By increasing the time, this network is broken and forms in the discontinuous state.
5. The isothermal solidification of assembly was completed at 1050 °C/60 min and the intermetallic compounds that formed was removed before this time, which includes nickel-rich boride and chromium-rich boride. Moreover, by removing the destructive phases that decrease the shear strength at 1, 15, 30, and 45 min, a shear strength of the assembly of 472 MPa at 1050 °C/60 min has been achieved near the base metals.

## Figures and Tables

**Table 1:** Chemical compositions of super-ferritic 446 and martensitic stainless steel 410 (wt.%).

Materials			Elements							
	Fe	Cr	Mn	Si	Ni	C	Mo	Nb	P	S
SFSS 446	Bal.	25	2	1	0.5	0.2	0.2	0.3	0.04	0.03
MSS 410	Bal.	12	1	1	0.1	0.15	-	-	0.04	0.03

**Table 2:** Chemical composition of nickel-based BNi-9 amorphous foil (wt.%).

Material	Elements					
	Ni	Cr	Si	B	C	Fe
BNi-2	Bal.	7	4.5	3.2	0.06	1.5

**Table 3:** Chemical composition of sigma phase at different conditions (wt.%).

Conditions	Phase	Elements					
As-received	Blocky	Fe	Ni	Cr	Si	Mo	Nb
	Plate	Bal.	0.37	60.1	0.04	0.02	0.05
1050 °C/60 min	Blocky	Bal.	0.34	58.19	0.03	0.01	0.02

**Table 4:** EDX analysis of various phases indicated in Figure 8 (wt.%).

Suggested Phase	Locations	Elements					
		Ni	Fe	Cr	Si	Mo	Nb
Solid solution γ NI-Based	ISZ_446_	Bal.	20.23	10.01	3.4	0.03	0.1
Solid solution γ NI-Based	ISZ_410_	Bal.	18.1	9.91	3.1	0.01	0.01

**Table 5:** EDX analysis of various phases indicated in Figure 9 (wt.%).

Suggested Phase	Figures	Elements					
		Ni	Fe	Cr	Si	Mo	Nb
Nickel-rich boride (Ni_3_B)	8	Bal.	5.43	3.96	0.76	0.01	0.01
Chromium-rich boride (CrB/Cr_2_B)	9	7.81	7.78	Bal.	1.21	0.02	0.01

## Data Availability

All data provided in the present manuscript are available to whom it may concern.
